# You Are *Not* Welcome! A Media Analysis of Risk Factors, Prevalence and Management of Free-Roaming Dogs in Iran

**DOI:** 10.3390/ani13142347

**Published:** 2023-07-18

**Authors:** Farshad Amiraslani

**Affiliations:** Independent Researcher, 53A, Millburn Road, Coleraine BT52 1QT, UK; fami8991@alumni.sydney.edu.au

**Keywords:** free roaming, free ranging, stray, dog, urban, Iran, online media, news articles, rabies, landscape, habitat

## Abstract

**Simple Summary:**

An increase in the number of free-roaming dogs and the related consequences (e.g., dog biting) has been observed across Iranian cities. So far, no clear scientific reasons for this increase were demonstrated, and a few existing domestic studies have only investigated the behaviours and frequencies of these animals in the cities. Based on online news articles, the on-the-ground reasons, challenges and dog-controlling activities mentioned by key urban and health authorities are examined in this paper. A scientific link is established between increased urbanisation, its features and its implications for dogs and humans.

**Abstract:**

History has witnessed a long-term relationship between humans and animals. Historical documents and modern findings prove that humans’ needs to use animals for companions or services are commonplace in many parts of the world, leading to the domestication of certain animals. Yet, modern societies have degraded many natural habitats for wildlife, confining them to small patches of landscapes or urban areas. Whether a domesticated/free-roaming animal or a wild species, their close contact with humans can create cumbersome situations for both species. This paper explores a link between online media content and on-the-ground efforts to manage free-roaming dogs as a rare case study. As indicated by news articles, the municipal costs of managing free-roaming dogs in Iranian cities have increased, and this can potentially derail the control of such dogs in the long run. This paper lays out pivotal factors for recent increasing human–animal encounters, which have led to many challenges (e.g., rabies) across cities in Iran. We show that some urban features (e.g., topography) can influence the presence and behaviours of free-roaming animals in the cities. The findings of this paper can be related to other developing countries where the plague of rabies is rising.

## 1. Introduction

### 1.1. A Short History of the ‘Human-Animal’ Intimacy Linkage

Foley and Gamble [1] describe the ecology of humanity’s social transitions. As one of these social transition eras, the so-called T5 evolutionary era (ecological intensification: 200–10 ka) portrayed an ‘energy-rich ecology through aquatic resources, cereal harvesting, hunting and domestication of animals’ (Table 3: [1]). It was a time when most of the population possibly lived in separate groups and landscapes without proper communication and social interactions, yet early settlers felt the need to initiate new forms of human communities and social interactions. The *H. sapiens* gradually made intergroup and regional social structures and networks, while the social system was dominated by resource ownership, defence and control (ibid.). The era was continued by a ‘fully sedentary, agricultural and ethnically complex world in the last 15 ka’ (ibid.).

Over the past millennia, humans have embraced the idea of domesticating animals for their benefit. The most notable cases are the domestication of goats (*Capra hircus*) that occurred 10,000 years ago [2]. A study reveals that dogs were independently domesticated several millennia ago in Eastern and Western Eurasia from distinct wolf populations [3]. ‘The earliest historical mention of domestic cats originates from Persia [now Iran] and dates to the sixth century CE, …women kept cats as pets, dying their fur, adorning them with jewellery, and letting them sleep in their beds’ [4]. There is also evidence of domestic cats (*Felis catus* L., 1758) from Kazakhstan, dating to 775–940 cal. CE (ibid.).

### 1.2. Contemporary World: Dominance of Human Species 

The contemporary world has changed dramatically. Over the past centuries, unprecedented population growth and resource utilisation have disrupted the equilibrium between humans and animals. Crowded urban areas and surrounding segmented forests and rangelands have created a chaotic world for millions of birds, mammals, insects, etc. A diverse range of wet markets, open/closed fish markets, local vendors of live animals, etc., have exacerbated this linkage to the point that a wave of health and hygienic risks in cities is imminent. Those earlier domesticated animals, known these days as pets, have approached our private life, but with associated risks. Common zoonotic diseases between humans and pets, especially dogs, have endangered our health and well-being (e.g., [5]). They can also be potential conservation threats to wildlife (e.g., [6,7]). 

The dysconnectivity between humans and dogs as companions in urban areas has culminated into an urban challenge due to the rising number of free-roaming dogs. The issue has become a cross-disciplinary subject intersecting veterinary science, urban planning, anthropology, epidemiology and other health-related sciences. The positive psychological and mental effects of this human–dog intimacy (e.g., [8]) could lead to serious health issues posed by free-roaming dogs (e.g., [9]). A range of humanistic adverse side effects (e.g., rabies), pertinent curing costs for humans and dogs and exerted costs on urban management have enticed city planners to re-evaluate their encounter with this growing public health concern worldwide. 

Contemporary growing urbanisation has made the relationship between humans and wildlife/domesticated animals much more special, but complicated [10,11,12,13,14]. We encounter and enjoy wildlife in our cities. We signify some animal species on our aircrafts (e.g., the oryx for Qatar Airways) and shelter them in our places for nesting, breeding and feeding [15]. Sometimes, though, we (un)intentionally injure or kill them (e.g., road accidents). 

Urbanisation has caused natural landscapes to become fragmented, disturbed and degraded. Few natural habitats have remained untouched and healthy in urban areas, and their fragile condition will worsen due to land use changes. The legacy of our toxic materials introduced to these territories in order to develop our built-up areas (e.g., cemented pavements), for our daily usage (e.g., face masks), or consequences of our habits (e.g., vehicle smoke) remains forever, with dire impacts on animals’ lives and health (e.g., Urban environment and cancer in wildlife; [16]). A strong link between urbanisation and the prevalence of pathogens in populations of free-roaming wildlife was established, mediated by an altered habitat structure and changes to the resource availability, leading to a low biodiversity and declining trends in species richness [13].

**Figure 1:**
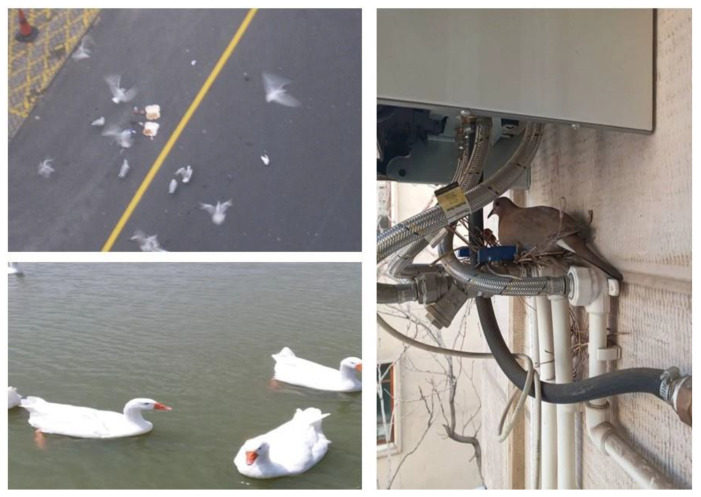
(Clockwise, from top left): Birds desperate for human leftovers on the street, Leeds—UK; nesting on a house’s boiler pipes, Tehran—Iran; foraging in a small pond within a university campus, Nanjing—China (all photos are from the author’s archives).

While at some point in the past, wildlife resided in large landscapes, currently, they are desperate to forage and explore resources outside of their original habitats, as we have changed these habitats to ‘urban’ areas (Figure 1). Even in the best situations, however, the unique settings of urban areas (e.g., buildings, highways) cause wildlife distraction due to lights or noises at night. These animals are thus being referred to as ‘urban adapters’, ‘urban exploiters’ or ‘urban avoiders’ [17]. 

Such an intricate and usually one-way human–animal relationship/impact has become a hot topic in many biodiversity and urban studies (e.g., [16,17,18]). Among these urban biodiversity topics is the consideration of free-roaming (free-ranging, stray) dogs or cats as a public health concern in many countries (e.g., [11,19]). Even the definitions and functions of each group within the same species differ. For instance, one researcher identifies the following three types of cats: ‘Confined, Free-Roaming and Feral’ [11]. While changed urban lifestyles and management have been critical factors in this trend, personal interests and circumstances could also be highlighted. The private ownership of pets, human treatment of these animals and their (un)intentional release afterwards are still debatable and controversial in animal welfare studies. Unowned pet cases are rising in societies. One research study found that the total urban unowned cat population in the UK could be 247,429 versus more than 10 million owned cats [20]. 

In urban realms, dogs, which are the most prominent and ubiquitous urban pets, are loved and hated [10]. Guard dogs and companion dogs have a relatively privileged position; in contrast, street and stray dogs are indications of a lower-status urban neighbourhood and are regarded as problematic urban subjects and virus transmitters, and the presence of ‘nuisance’ dogs can cause the eviction of residents, etc. [10]. Global reports prove that there are high public risks as a result of close contact or unprovoked attacks by free-roaming dogs [21]. Free-roaming animals are especially prevalent in the Global South. Reports indicate the correlation between these animals and the rising number of rabies cases in urban and suburban areas (e.g., Afghanistan: [5]; India: [6]).

### 1.3. Research Logic: The Problem Statement

Most studies on free-roaming dogs in the cities only address demographic, physiological, epidemiological and behavioural aspects. Less attention has been paid to fundamental urban reasons for creating suitable environments and conditions for such dogs to adapt to the cities. 

This research intends to fill this gap by focusing on Iran as a case study. There has been a surge in the number of free-roaming dogs and the related consequences (e.g., rabies) in urban and suburban areas in Iran (e.g., [22,23,24,25]). According to the Ministry of Health in Iran, animal bite cases increased from 35 per 100,000 people in 1987 to 177 per 100,000 people in 2016 [22]. As such, more general public and official concerns rose to inquire about the increasing dog attack incidents, processes to tackle these growing encounters and possible solutions to eradicate them. 

The goals of this research are three-fold as follows: first, to explore the spatial distribution and frequency of free-roaming dogs in various parts of Iran; second, to evaluate typical narrations of news articles to inform the public about free-roaming dogs (views on dog impacts); and third, to assess post-capturing operations publicised in online news platforms.

As rare research for Iran, this paper reviews the less-told challenges regarding existing domestic dog-controlling facilities and activities based on relevant news articles published in an online Iranian news platform. For instance, this paper explains the relationship between extensive land use changes in Iran that have consequently led to the removal of natural wildlife corridors between urban and suburban areas. This paper bases its discussion and explanations on qualitative information from news articles published in Persian. So far, no particular study or investigation has covered the possible spatial reasons for this surge in the number of free-roaming dogs in Iran, though information on the free-roaming dog population in Iran is very poor [26]. Existing domestic studies have only evaluated these rising concerns from epidemiological and medical points of view (e.g., [24,25]). 

## 2. Data and Methodology

‘The popular press (defined as print or online news articles meant for a general audience, as opposed to technical or trade publications) is an important source of information’ ([27], p.144). Media (print or online) may influence people’s understanding and views or inform people about topics they do not personally experience [27]. 

News article text or content analysis could be a practical approach to gathering on-the-ground and reliable data and information for many research topics and subjects. The content analysis could be regarded as a randomised sampling as no predetermined sampling methods, geographical areas or guidelines are utilised, though the original content could be biased. Also, they could cover much larger geographical regions and include a more diverse group of beneficiaries or stakeholders who are involved. Data gathering and analysis techniques are cost-effective, more straightforward and fast. A similar content analysis approach was used to comprehend the messages and outlines of wildlife news published in Iranian newspapers over a 7-year period [15]. Also, we found a global content analysis of media regarding free-roaming cats [27]. 

A popular Iranian online news broadcasting platform (ISNA: Iranian Students’ News Agency) was examined here. It is an online news platform that attracts a diverse range of audiences. News articles from this platform, published in Persian, on ‘free-roaming dogs’ covering three months (March–May 2023) were collated. Each news article was individually screened, and key messages and other features (e.g., stakeholders) were extracted. 

The dataset constituted 27 news articles covering several cities across 12 provinces (out of 31 provinces) as follows: East Azarbaijan (Tabriz), Mazandaran (several cities), Tehran (Tehran and suburban areas), South Khorasan (Birjand), Isfahan (Ardestan), Alborz, Qom (Dastjerd), Fars, Kerman (Narmashir), Khorasan Razavi, Khoramabad (Boroujerd) and Zanjan.

Although the selected news articles may not constitute a large dataset, the dataset is deemed a suitable proxy for revealing rising public concerns regarding free-roaming dogs across many provinces in the country. In this research, the dog-related challenges of the remaining provinces were also explained using other Iranian papers published on this subject. Discussions were compared with international findings and reports. 

## 3. Results 

### 3.1. Spatial Distribution and Frequency

The spatial distribution of free-roaming dogs reveals that most large provinces are affected by this issue. In our research, the case studies covered cities located in humid, semi-humid and dryland areas. Regardless of their geographical and climatic differences, some cities in each province reported similar trends in rising concerns about the high number of dogs in the streets. For instance, one news article refers to the recent annual 600% increase in street dog numbers. Some news articles indicate that ‘street/free-roaming dogs’ were among the top five municipality complaints raised by local people. Nevertheless, some news articles refer to this rise being due to foodstuff availability in streets and deliberate dog feeding by some citizens.

### 3.2. Views on Dog Impacts 

**Figure 2:**
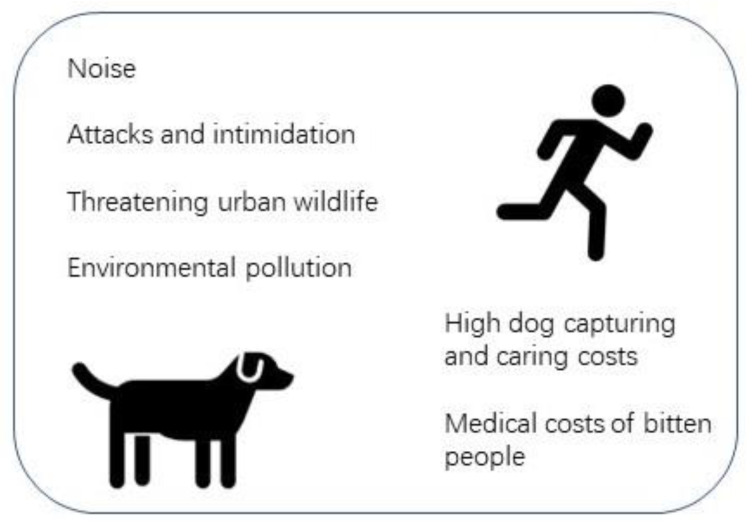
Infographic of causes of concerns reflected by news articles in an Iranian online news platform.

Figure 2 illustrates the principal issues reflected in news articles in an Iranian online news platform (ISNA) during a 3-month timeframe in 2023. The language and tones of the narrators or reporters of all news articles regarding free-roaming dogs are negative. One news article points out a municipality in a small city and its problems that needed particular attention due to the high maintenance costs associated with clinics for caring for dogs. Larger cities also raised concerns about such costs being unpredicted in their annual budget. One news article refers to one child’s death and several hundred casualties resulting from dog biting incidents. 

**Figure 3:**
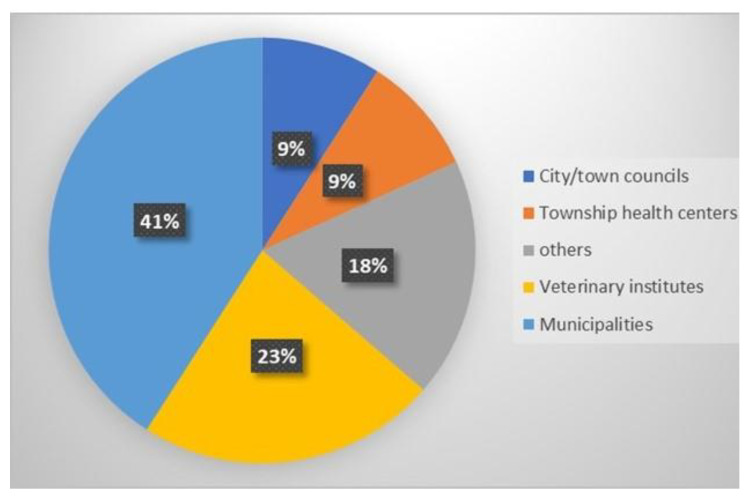
Share of organisations commenting on free-roaming dogs in online news articles.

The impacts of free-roaming dogs were mentioned mainly by urban officials. A diverse range of health, financial and administrative concerns were reported. It was found that most of the news articles were reported by municipalities (41%), followed by veterinary institutes (23%) (Figure 3). In Iran, municipalities encompass specific operational ‘Waste Management’ departments that collect/recycle solid wastes on the street. Now, they have also been tasked to manage free-roaming animals. 

The positive side is that these two entities (municipalities and veterinary institutes) are home to experts with relevant knowledge and qualifications regarding environmental public health and zoology. Nonetheless, no NGOs, policymakers or research organisations were involved in preparing, commenting or reporting free-roaming animals in online news. 

### 3.3. Post-Capturing Operations/Clinics

The news articles did not mention how dogs are captured, though the procedure necessitates physical contact and trapping. Several news articles stated the presence of newly constructed caring houses (dog clinics) for such dogs in many cities, ranging from 0.3 to 8.5 ha in area, as a combination of buildings, open yards and veterinary clinics. These dog clinics have particular kitchens, separation spaces, treatment rooms, etc. The news articles also refer to stakeholders being involved, including those who directly capture dogs from the streets, veterinarians and clinics’ cleaners. Several operations are implemented after capturing free-roaming dogs, outlined as follows:

- Segregation of male/female dogs.
- Early medical examination: Before being mixed with other group members, early medical interventions provide information about the body condition, weight, possible scars or other relevant health evidence about the new arrivals.
- Sterilisation: Predominant messages of news articles highlighted the need for current operations to sterilise dogs after capturing them. The process needs human expertise, medical facilities and other costly treatment procedures.
- Vaccination.
- Treating and caring for disabled/ill animals.

**Figure 4:**
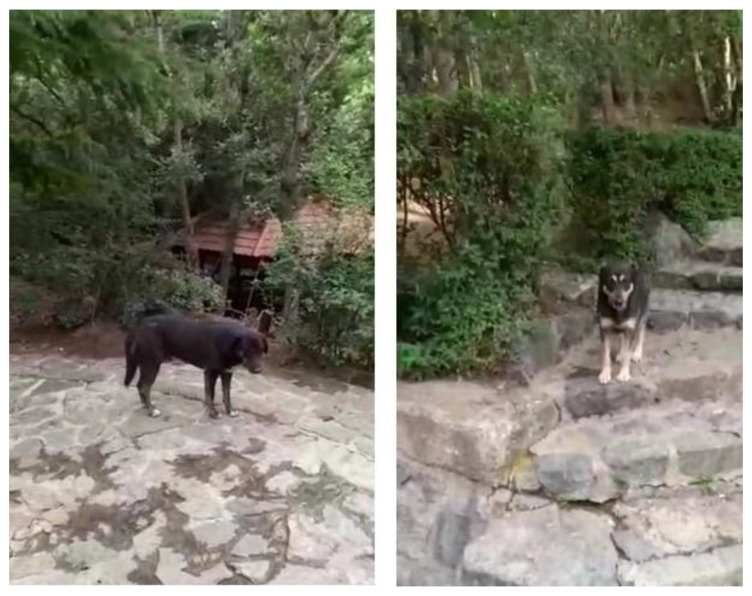
Vaccinated free-roaming dogs spotted in a top Tehran park—Jamshidieh Park (photos taken by the author).

Given the limitation of space and finance for caring, dogs are usually released to nature after being vaccinated/sterilised, though many will return to cities for food and shelter afterwards (Figure 4).

## 4. Discussion

### 4.1. Elaboration of Dog Clinics/Shelters in Iran

Our pool of news articles published on free-roaming dogs in Iranian cities reveals a growing concern among all urban and non-urban authorities. The issue has become spatially more diverse, from a limited number of provinces years ago to covering almost all provinces (Section 3.1).

The concept of publicly managed and funded dog clinics/shelters is new in Iran. Earlier attempts to capture and shelter free-roaming dogs in Iran were made based on people’s interests and funds. People erected a handful of suburban shelters with no governmental support. Nevertheless, given the rising public health issues in many cities, mayors or other authorities started to accept that a large-scale movement is needed (see Section 3.2). 

Based on reliable news articles, our research elaborates on some of the critical medical and health concerns (Section 3.3). For instance, a news article mentioned the segregation of male/female dogs. Although there is no national research on this issue, one study showed a significant male-to-female ratio (3.2/1) among the free-roaming dog population in Kerman City [26]. Therefore, such segregation assists in better controlling mating and keeping an average balance among dogs in the shelter. 

Also, the news articles generally mentioned the sterilisation of dogs as a key activity in their centres. This procedure guarantees the control of the future propagation of these dogs. Elsewhere, research conducted in Kerman City suggests that more female neutering coverage can hugely decrease the population size of free-roaming dogs [26].

### 4.2. Rising Concerns in Iran

The news articles refer to major concerns described in Figure 2. Here, we elaborate on them in more detail as follows:

- Increasing incidences of dangerous and unprovoked dog attacks in the streets: Domestic research conducted in a city in central Iran revealed that over 92% of dog attacks were unprovoked [28]. Free-roaming dogs and their associated diseases (e.g., rabies) could be regarded as both rural and urban challenges. It was found that rabies has two epidemiological cycles, which are an urban cycle and a sylvatic cycle [29].
- Human health concerns of dog bites (e.g., rabies): It was shown that the prevalent rabies cases in Iran occur due to dog biting (over 95% of rabies cases) [30].
- Threatened outdoor activities of families: A recent 30-year global study shows that there is a correlation between the participation of people (especially men) in outdoor activities and the likelihood of biting incidences and infection with the rabies virus [31]. The physical exposures of humans to free-roaming dogs in outdoor environments increase the risks of dog attacks and biting even without any animal annoyance.
- Increasing municipalities’ costs of controls: All news articles highlight the increasing costs of controlling, curing and keeping free-roaming dogs. The news articles refer to the costs of running dog clinics and their personnel, dog vaccines, part-time veterinarians’ expenses, medicines, etc. These dog clinics are new entities, and many cities do not have the human, land and technical resources to establish them.
- Impacts on water and soil resources: The news articles did not explain the impacts, nor was any research found on this matter in Iran. Nevertheless, the urination and defecation of dogs could add hazardous materials to the environment, pollute soil and water resources and affect human health (e.g., [32,33]).
- Threats to wildlife health and survival: Although the news articles did not address this important point, there are numerous global reports on the impact of free-roaming animals on wildlife (e.g., [7]).
- Noise pollution: The excessive barking and soiling of community spaces are enumerated as usual free-roaming dog behaviours causing a public nuisance [34].

### 4.3. Influencing Factors

Urbanisation is regarded as one of the potential factors contributing to increasing rabies cases (e.g., [35]). The recent trends in biting incidences in Iran reveal that most animal bites occur in urban areas, contrasting with national reports [36]. The analysed news articles referred to some, but not *all*, urban features concerning rabies cases in Iran. In the following list, the author explains the most important urban characteristics causing rabies cases:

- Availability of foodstuff in streets and deliberate dog-feeding (mentioned by news articles): The rising inclination of people to feed animals has become a severe challenge for urban authorities in Iran. There is a sign stating ‘no feeding to animals’ in almost every corner of greenery spaces and parks. Nevertheless, some people still insist on following their passion for feeding animals. This behaviour could encourage free-roaming animals to stay, breed and expand their territories in the cities. The research conducted shows that the likelihood of sighting an ideal or overweight dog in the city was 14.9 times higher than in the suburbs in Kerman City, indicating a much higher food availability for dogs in the city [26].
- No facilities for surveillance and monitoring of free-roaming dogs (mentioned by news articles): This reason is pertinent to the lack of overall animal/pet keeping and management systems and guidelines, including in zoos in Iran [15]. Also, small cities nationwide encompass various types of local illegal wet/bird markets without the proper human health and animal hygiene systems.
- City topography (mentioned by news articles): Iranian research emphasises the regional and geographical heterogeneity of rabies cases in Iran [29]. The news articles mention this factor without any further description, though this is one of the critical parameters in urban areas favouring free-roaming animals, including dogs. For example, Tehran, which is known as a valley city, is extended on the hillslopes of adjacent mountainous regions. Such topographical features offer various options to animals for breeding, hiding, roaming and escaping. Also, many districts in the city possess polluted water channels, favouring free-roaming animals. This latter issue was found to be relevant in transmitting the rabies virus in Arequipa (Peru) [37].
- Lost transitional zones between the urbanised areas and the surrounding forested/mountainous areas (mentioned by news articles): These transitional zones that are adjacent to cities could provide open spaces (buffers) for animal/wildlife roaming and movement without being interfered by human activities. For instance, in one of the cities in Central Iran (Kerman), vacant lots located in the older parts of the city with ruins of abandoned old buildings had the largest number of free-roaming dogs [26]. Nevertheless, such a factor was not deemed relevant in a study conducted in Argentina [38].
- Urbanisation and unbalanced relationships between wildlife and the surrounding territories: The conditions changed after rapid urbanisation started in Iran in the 1930s when urban populations increased due to rural–urban migrations. The oil-funded urbanisation in Iran was initiated earlier than in many other neighbouring countries. A massive flux of rural–urban migration created a chaotic condition for the then-small city [39]. This unprecedented urban sprawl resulted in an unequal distribution of urban amenities, crimes, improper solid waste and sewage management, among others. These bottlenecks favour many free-roaming animals.
- Emerging new urban slums: This is particularly relevant to most Iranian cities, as new illegal settlements and urban slums were developed due to rising land and housing prices in cities. It takes time for these areas to become recognised by municipalities as a city (to receive urban facilities and services) or by other governmental organisations as legal entities for land registration and utilities. These areas lack proper hygienic arrangements, including bin collection. Research shows that the abundance of unowned cats is increased in more deprived urban areas with a higher human population density [20]. Therefore, such a chaotic situation makes these areas perfect places for encountering free-roaming dogs/cats. Global research establishes a link between certain infectious diseases (e.g., cholera) and slums due to poor infrastructure and a lack of access to safer water and better sanitation [14].
- Changed lifestyle and behaviours: Culturally and religiously, keeping pets, especially dogs, is not a common custom in Iran, though this case was also mentioned for Afghanistan [5]. Dogs were always key working partners for rural people, including farm owners, herders and farmers in Iran. Currently, more and more Iranians live alone, which may also be a factor in choosing to live with a pet. Also, as some unofficial reports suggest, younger Iranian generations prefer to keep pets rather than raise children at home. Nevertheless, this does not necessarily indicate that these people will keep their pets forever. Given the rising living expenses in cities, including the costs of pets’ health care and food, there is a high chance that pet owners will leave dogs in surrounding areas. Moreover, over half of the owned dogs in Iran have not been vaccinated annually [25], which can increase the health risks for animals and humans.

In addition to urban characteristics, the other factors affecting the spread and control of rabies are as follows:

- The COVID-19 pandemic: For most of the two years during the COVID-19 pandemic, like other countries, many activities of service-providing organisations, including municipalities, were halted or reduced in Iran. Such a gap in controlling urban pests and maintaining health orders were also affected, although there were rumours regarding the correlation between free-roaming animals and COVID-19 at that time. Nevertheless, a few cities in Iran recorded lower levels of rabies incidence during the COVID-19 period due to fewer outdoor activities or the avoidance of medical services for rabies treatment (e.g., [28]).
- Ecologically, free-roaming dogs exhibit broad temporal and spatial plasticity, broad distribution and population explosion and tolerance to environmentally different areas [40]. A report shows that some dogs in Kenya travel up to 24 km daily [34]. Such adaptabilities make controlling free-roaming dogs more difficult.
- The seasonality of rabies incidence must be highlighted. A meta-analysis of published research on rabies incidences in Iran shows that dog biting mainly occurred in the ‘spring’ seasons [29]. In China, however, a study revealed that ‘August’ was the peak month for rabies in 29 years [35]. Another Iranian research study also attributes some incidences to long-term drought and its associated lack of water and food in natural habitats in the Kerman province, located in the dryland areas of Iran [28]. High temperatures are positively correlated with the risk of rabies incidence, as dogs are more irritable and more likely to bite people in hot weather [41]. Moreover, proper national/provincial vaccination policies and educational programmes could effectively reduce rabies cases (e.g., China: [41]).

The above-mentioned dog-mediated issues raised by Iranian news articles could be observed in almost all cities in Iran, but Tehran, as a capital city, has drawn attention to public health. The city has the largest population in the country, which makes the city more vulnerable to public health challenges. The quality of life in Tehran is uneven and unjust, and each district suffers from urban inequality, inefficiency and/or deficiency [39]. In particular, Tehran is the most exposed location to free-roaming dogs for several reasons. Tehran is surrounded by expansive barren lands and non-built areas, which makes it the best living and hiding option for such dogs. The city has the densest entertaining and eating places that provide free food waste for free-roaming animals (e.g., rats and dogs). For decades, the rat plague has been common in Tehran. As such, the Tehran municipality has tried to identify the hotspots of groups (usually inside uncovered street water ditches or garbage sites) and eradicate them using poison baits. An Indian report shows the presence of free-roaming dogs close to garbage bins, predominantly within a 20 m radius in urban settings [42]. Finally, Tehran has one of the largest greenery spaces and parks in Iran, and these places provide food (by means of people feeding dogs) during the day and shelter at night for free-roaming dogs. 

### 4.4. Worrying Trends of Rabies Occurrences in Iran

Worldwide, rabies still kills about 60,000 people a year, varying among countries and population age groups [43]. Dog vaccination is the most effective measure of rabies control [43].

In Iran, rabies is endemic, and old documents prepared by Iranian scholars such as Avicenna (Ibn Sina, 980–1037 AD) and others have described the transmission and treatment methods of rabies [44]. Modern scientific research on rabies and controlling measures have been followed in Iran since 1924, upon the establishment of the Pasteur Institute in Iran [25]. The rabies disease was fully controlled in Iran by 1977 [30]. 

Over the past decades, the frequency and distribution of rabies in Iran have changed, reflecting the social and economic conditions of people. The mortality rate due to rabies decreased from 0.9 per million people in the 1980s to 0.02–0.03 in recent years in the country [29]. Recent urban rabies cases are rising in Iran [28,36], and the new surge highlights the complexity of unpredictable risks in contemporary lifestyles and societies’ desires. 

### 4.5. Raising Awareness of Free-Roaming Dogs in Iran

As indicated in the previous sections, an online news platform regularly released news on this matter during our study period. Such dissemination of information is necessary for all people, especially vulnerable people, such as disabled or senior citizens, due to fewer mobility and self-defence opportunities. Equally important, children and pregnant women must be informed as they may show similar weaknesses when encountered by free-roaming animals. The problem is that only some of these affected groups can access such news platforms, and thus, urban authorities must convene workshops, TV programmes and school sessions for further effectiveness. Nevertheless, a previous study on publicising wildlife-related news in Iranian newspapers highlights the general low frequency and ineffective news dissemination [15].

## 5. Conclusions

Here, for the first time in Iran, we utilised a news articles analysis for covering free-roaming dogs in the streets. Our study encompasses broader geographical areas (12 out of 31 provinces) and a diverse pool of commenting/involved stakeholders. It reveals the growing concerns regarding the frequency and impacts of increasing free-roaming dogs and subsequent rabies in certain cities in Iran. The importance of the information used in this research is that these news articles cover the most recent reliable concerns of public and governmental organisations regarding the rising number of dog attacks, health-related challenges and rising costs of protective and treatment operations in urban/suburban areas.

It was beyond the scope of this paper to assess the communicative language or tone of Iranian news articles. Nevertheless, the Iranian news articles intend to present these animals’ negative image, perhaps for public health.

We also highlighted the urban features that influence the presence and behaviours of free-roaming animals in the cities (e.g., topography, slums, etc.). These features have great potential to be revisited in other urban contexts, as these studies offer solutions to resolve diseases linked to free-roaming animals. The subject could also be revisited by others to understand the whole cycle of this challenge in urban and associated social concerns. 

Current scant datasets regarding the free-roaming animals in Iran downgrade many conclusions and findings to speculations. Data gathered at finer spatial resolutions (e.g., citizen science techniques) and/or targeted ground-based data can improve our understanding of the dynamics and behaviours of free-roaming animals in our cities.

Finally, despite the negative image of dogs (free-roaming types) in this research and other similar papers, we must emphasise the positive roles of tame dogs who are real companions for supporting human mental health, guiding blind people or assisting disabled/deaf people or people who suffer from dementia. They remain our friends during long-term hardships or illnesses when many people leave or cannot support us.

## Data Availability

No data is available.
